# Glycated hemoglobin A1c level on the day of emergency surgery is a marker of premorbid glycemic control: a retrospective observational study

**DOI:** 10.1186/s12871-018-0641-2

**Published:** 2018-11-30

**Authors:** Mai Hokka, Moritoki Egi, Satoshi Mizobuchi

**Affiliations:** Department of Anesthesiology, Kobe University Hospital, 7-5-2 Kusunoki-cho, Chuo-ku, Kobe City, 650-0017 Japan

**Keywords:** Emergency surgery, Premorbid glycemic control, Glycated hemoglobin A1c

## Abstract

**Background:**

Current international guideline recommends to maintain blood glucose level ≤ 180 mg/dL in acute ill patients, irrespective of presence of premorbid diabetes. However, there are studies suggested that optimal acute glycemic control should be adjusted according to premorbid glycemic control in patients with chronic hyperglycemia. Accordingly, to obtain the information of premorbid glycemic control would be relevant. However, the HbA1c level on the day of the emergency operation (HbA1c-ope) might not be useful as a surrogate of premorbid chronic glycemic control, since glucose metabolism can be affected by inflammation, severity of illness and surgical invasion.

**Methods:**

We hypothesized that HbA1c-ope reflects pre-morbid glycemic control. To assess this hypothesis, we conducted a single-center retrospective observational study to assess the association between HbA1c-ope and HbA1c level measured within 30 days before the operation (HbA1c-pre). We screened patients who had been admitted to the ICU of our hospital after emergency surgery during the period from January 2008 to December 2016. Patients in whom both of HbA1c-ope and HbA1c-pre were measured were included in this study. We compared HbA1c-ope and HbA1c-pre using the paired t-test. The correlation between the two HbA1c measurements was assessed using Pearson’s correlation coefficient. Its agreement was assessed using the Bland-Altman approach with 95% confidence intervals.

**Results:**

We included 48 patients in this study. The mean value of HbA1c-pre was 6.3%, which was not significantly different from the mean value of 6.2% for HbA1c-ope (*p* = 0.12). There was a significant correlation between HbA1c-pre and HbA1c-ope (r^2^ = 0.70, *p* < 0.001). The mean difference between two HbA1c measurements was 0.12% (95% CI: -0.03% to 0.27%). The limit of agreement ranged from − 0.9% to +1.1%.

**Conclusions:**

We found that there was a significant correlation between HbA1c-ope and HbA1c-pre. Our findings suggest that HbA1c-ope can be used to estimate previous glycemic control with an acceptable degree of accuracy, enabling personalized glycemic control in the perioperative period.

**Electronic supplementary material:**

The online version of this article (10.1186/s12871-018-0641-2) contains supplementary material, which is available to authorized users.

## Background

According to the results of NICE-SUGAR trial [1] and subsequent meta-analysis [2], international guideline recommends to maintain blood glucose level ≤ 180 mg/dL irrespective of presence of premorbid diabetes [3]. However, there have been a number of studies showing that the association of acute glycemic control with worsened outcomes was altered in patients with diabetes mellitus [4]. There was an inverse relationship of average glycemic control in the ICU with mortality outcomes in patients with high HbA1c but not in patients with low HbA1c [5]. These observations lead to the hypothesis that glucose concentrations, which might be considered safe and desirable in other ICU patients, might be undesirable and optimal blood glucose concentration might be higher in critically ill patients with the presence of chronic hyperglycemia. In fact, there were sequential-period exploratory studies which reported that liberal glucose control (target glycemia: < 14 mmol/L) appears to attenuate glycemic variability and may reduce the prevalence of moderate-severe hypoglycemia in compared with standard care (< 10 mmol/L) in acute ill patients with chronic hyperglycemia (HbA1c ≥ 7.0%) [6, 7].

Considering the above findings, it might be important to determine the degree of chronic hyperglycemia in patients receiving an emergency operation and subsequent intensive care [8]. However, in the perioperative period, secretion of stress hormones and cytokines may be influenced by inflammation, severity of the condition and surgical invasion, which may affect glucose metabolism and result in glycemic derangement [9]. Thus, there may be concern to use the HbA1c level on the day of the emergency operation (HbA1c-ope) as a surrogate of premorbid chronic glycemic control, since such an alteration of glycemic control in an acute illness setting may also affect HbA1c level [9]. It is unfortunate, there has been no study carried out to determine HbA1c-ope reflects pre-morbid glycemic control in such patients.

Accordingly, we hypothesized that HbA1c-ope reflects pre-morbid glycemic control. To assess this hypothesis we conducted a single-center retrospective observational study to assess the association between HbA1c-ope and HbA1c level measured within 30 days before the operation (HbA1c-pre).

## Methods

### Design

This single-center retrospective observational study was approved by Kobe University Hospital Ethics Committee (Approved No. 1587). The committee waived the need for informed consent for studies involving the use of a database.

### Setting and participants

We screened patients who had been admitted to the ICU of our hospital after emergency surgery during the period from January 2009 to September 2015. Patients in whom both of HbA1c-ope and HbA1c-pre were measured were included in this study.

### Data collection

We collected data from electronic medical records for patients’ characteristics including age, sex, American Society of Anesthesiology (ASA) physical status classification, prior diagnosis of diabetes mellitus (DM), insulin dependency, operation time, anesthesia time, and type of surgery. We also collected both of HbA1c-ope and HbA1c-pre. All HbA1c levels was measured in the same laboratory (AdamsA1c HA-8181**,** ARKRAY, Japan).

### Statistical analysis

The results are shown as means with standard deviation or *n* (%). We compared HbA1c-ope and HbA1c-pre using the paired t-test. The correlation between two HbA1c measurements was assessed using Pearson’s correlation coefficient. Its agreement was assessed using the Bland-Altman approach with 95% confidence intervals. To assess possible factors associated with bias of HbA1c measurements, we further performed linear regression analysis to assess the association with the difference between two HbA1c measurements. We also collected detailed information for patents with a bias larger than 10% of HbA1-pre.

Data were reported in accordance with the *Strengthening the Reporting of Observational Studies in Epidemiology* (STROBE) guidelines [10]. We used R to perform statistical analysis. A *P* value < 0.05 was defined as a statistically significant difference.

## Results

### Patients’ demographics

We included 48 patients in this study (Additional file 1). Patients’ demographics are shown in Table 1. The mean age of the patients was 68 years. The ASA physical status classification was 3E or 4E in approximately 80% of the patients. There were 21 patients (43.8%) with pre-diagnosed DM. Cardiovascular surgery was performed in the majority of patients (75%). The hospital mortality in study cohort was 8.4%.

**Table 1:** Patients’ demographics

Age (years)	68 ± 12
Male. *n* (%)	29 (60.4%)
ASA PS. *n* (%)	
2E	10 (20.8%)
3E	31 (64.6%)
4E	7 (14.6%)
Presence of diabetes mellitus	21 (43.8%)
Insulin treatment	8 (16.7%)
Operation time (mins)	249 ± 134
Anesthesia time (mins)	306 ± 142
Operation categories	
Cardiovascular surgery	36 (75%)
Gastrointestinal surgery	6 (12.5%)
Thoracic surgery	3 (6.3%)
Other surgery	3 (6.3%)

Values are means±SD or n(%)

ASA PS: American Society of Anesthesiology physical status

#### HbA1c measurements on the day of the emergency operation and within 30 days before the operation

**Fig. 1:**
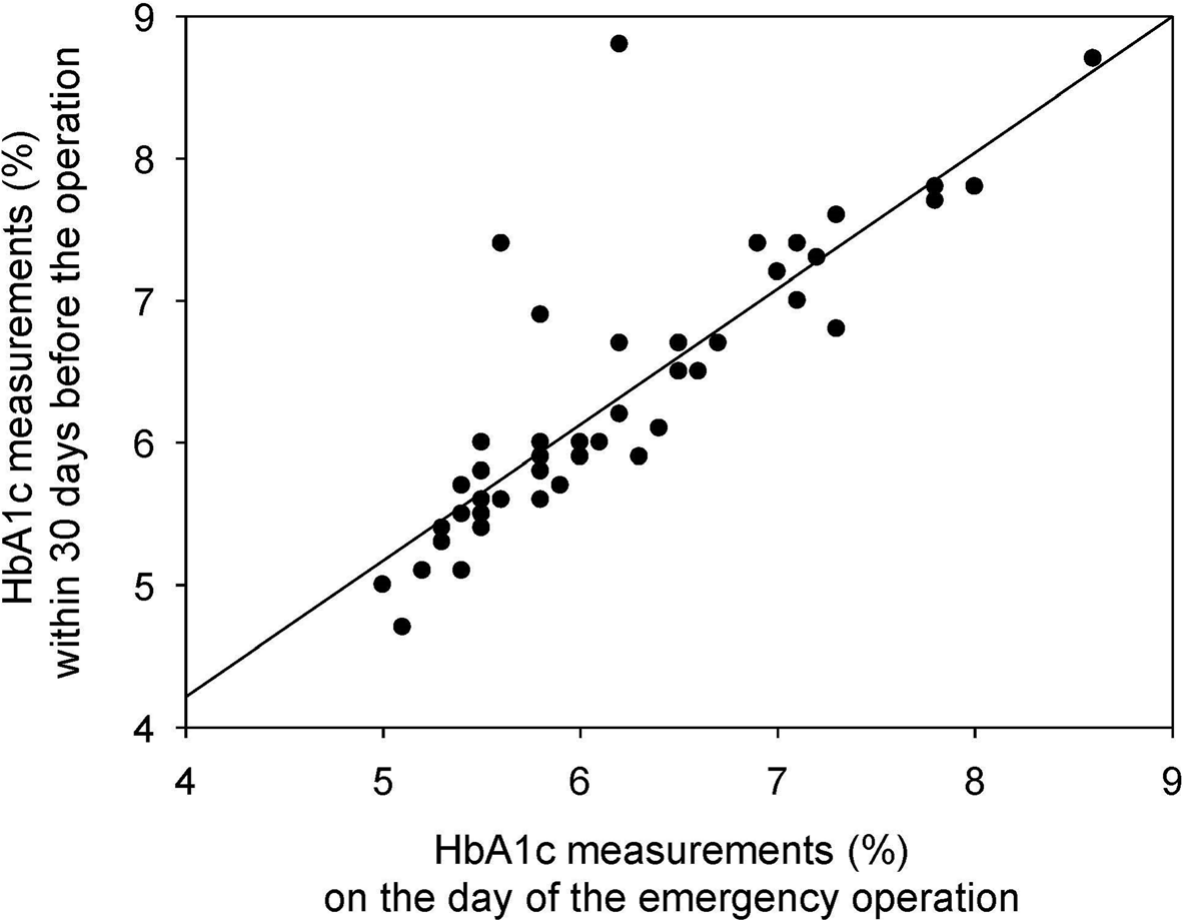
Correlation between HbA1c measurements on the day of the emergency operation and within 30 days before the operation. This figure shows the correlation between the two HbA1c levels. The vertical axis shows HbA1c measurements within 30 days before the operation (HbA1c-pre), and the horizontal axis shows HbA1c measurements on the day of the emergency operation (HbA1c-ope). There was a significant correlation between preHbA1c and opeHbA1c (r^2^ = 0.70, *p* < 0.001). Straight line indicated the regression line ([HbA1c-pre] = 0.39 + 0.96* [HbA1c-ope])

**Fig. 2:**
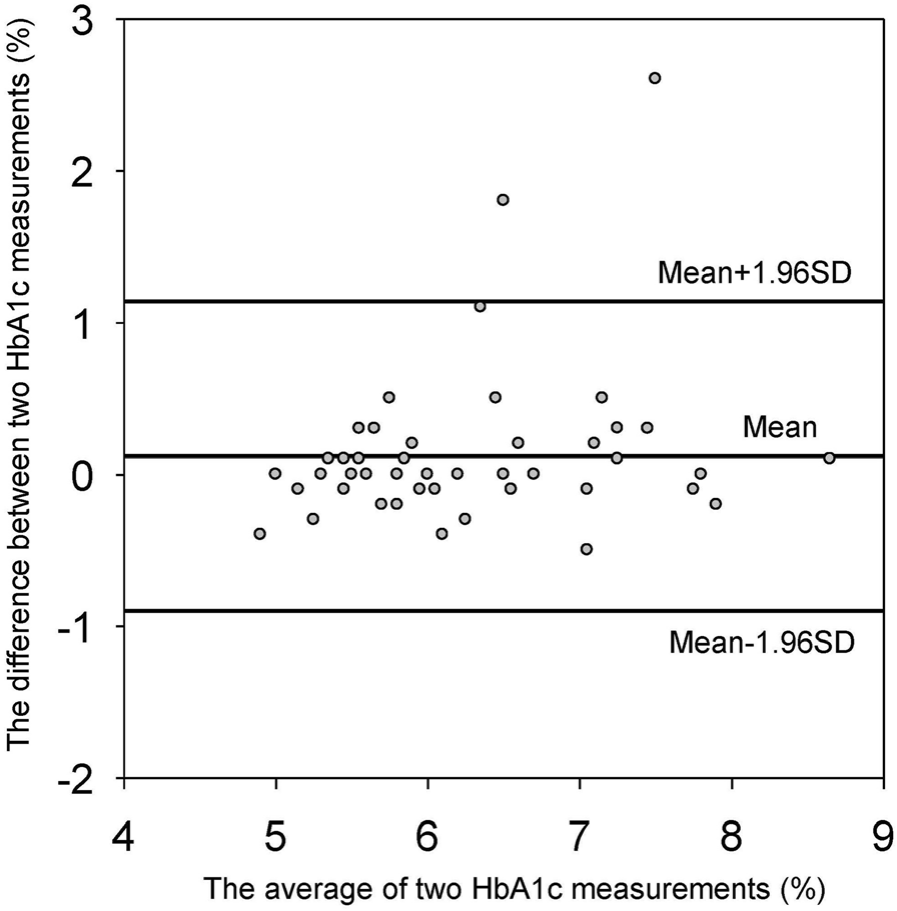
Comparison of HbA1c measurements on the day of the emergency operation and within 30 days before the operation using a Bland-Altman plot. This figure shows a comparison of HbA1c measurements on the day of the emergency operation and within 30 days before the operation using a Bland-Altman plot. The vertical axis shows the difference between the two HbA1c measurements, and the horizontal axis shows the average of the measurements. The middle vertical bar indicates the average difference between two measurements (+ 0.12%).The upper and lower bars indicate the limits of agreement (-0.9% and +1.1%)

HbA1c-pre was measured 11 days before the operation on average. The mean HbA1c-pre value was 6.3%, which was not significantly different from the mean value of 6.2% for HbA1c-ope (*p* = 0.12). There were 11 patients with HbA1c-pre > 7%, and all them were pre-diagnosed as DM. There was a significant correlation between HbA1c-pre and HbA1c-ope (r^2^ = 0.70, *p* < 0.001) (Fig. 1). Figure 2 shows a Bland Altman method plot for the two HbA1c measurements. The mean difference between HbA1c-pre and HbA1c-ope was + 0.12% (95% CI: -0.03% to 0.27%). The limit of agreement between the two measurements ranged from -0.9% to +1.1%.

#### Possible factors associated with the bias of two HbA1c measurements

To determine the possible factors associated with the bias of two HbA1c measurements, we assessed the correlations of its bias with demographics (Table 2). The bias of two HbA1c measurements did not have any significant association with age, sex, ASA physical status classification, operation time, anesthesia time, type of surgery (cardiac surgery vs non-cardiac surgery) or duration between the two HbA1c measurements. The bias of two Hba1c levels was significantly associated with insulin treatment before the operation (*p* < 0.001).

**Table 2:** Results of linear regression analysis to determine the associations between factors and change in HbA1c

Factors	r^2^	*P*-value
Age	0.04	0.19
Sex	0.0003	0.91
ASA PS	0.001	0.81
Insulin treatment	0.35	< 0.001
Operation time	0.02	0.29
Operation categories (cardiovascular surgery vs others)	0.006	0.59
Duration between measurements of the two HbA1c levels.	0.02	0.36

ASA PS: American Society of Anesthesiology physical status

There were 3 patients with a bias larger than 10% of pre HbA1c (Fig. 2). HbA1c-pre of the three patients were more than 6.5%. Two of them had required large transfusion of red blood cells before measurement of HbA1c-ope (11 units for one patient and 6 units for the other patient). The other patient received intensive insulin therapy and required emergency surgery 24 days after measurement of HbA1c-pre.

## Discussion

### Key findings

In this study, we found that there was a significant correlation between HbA1c measured on the day of the emergency operation and those measured within 30 days prior to the operation. We also found that 3 (6%) of the 48 patients had a large bias (> 10%) between the two HbA1c measurements. Such a bias might have been caused by the red cell transfusions or intensive insulin therapy between measurements of two HbA1c. This is the first study to assess the utility of HbA1c level at the day of an emergency operation as a surrogate marker of premorbid glucose control, thus further discussion should be required.

### Comparison with prior studies

There has been no other study carried out to determine whether HbA1c-ope is a useful surrogate marker of premorbid glycemic control before emergency surgery. However, there has been two studies in which such a hypothesis was assessed in a critical illness setting and an emergency department setting.

Luethi et al. conducted a retrospective study to assess the relationship between HbA1c level on ICU admission and premorbid HbA1c level. In that study conducted in 69 critically ill patients, a strong correlation was found between HbA1c levels on ICU admission and premorbid HbA1c levels (*r* = 0.89; *p* < 0.001). They also reported that red blood cell transfusion was significantly associated with bias of HbA1c levels [11]. Thakker et al. conducted a retrospective study in 589 adult patients with no known history of diabetes in whom HbA1c levels were measured in an emergency department. They evaluated intra-patient differences in HbA1c levels measured in the emergency department and after recovery from acute illness. They found that the HbA1c levels was highly correlated (r^2^ = 0.83) and they concluded that HbA1c level is not substantially affected by acute illness. They did not assess factors that influence the bias of HbA1c levels [12].

Those two studies showed that HbA1c levels are not altered by the onset of critical illness and that HbA1c levels at admission to the ICU or emergency department can be used to estimate chronic glycemic control and can serve as a guide for acute glycemic therapy. Although our study was conducted in patients who required emergency surgery, our findings are in line with the results of those two studies.

#### Interpretation

There were studies that hyperglycemia was associated with a higher risk of death in patients without diabetics or with low HbA1c, but not in patients with high HbA1c [5, 13]. Additionally, in a recent retrospective multicenter observational study, chronic pre-morbid hyperglycemia was shown to increase the risk of hypoglycemia and to modify the association between acute hypoglycemia and mortality [14]. These observations lead to the hypothesis that liberal glucose control (target glycemia: < 14 mmol/L) might be optimal in critically ill patients with the presence of chronic hyperglycemia [6, 7]. Currently, LUCID trial (the Liberal GlUcose Control in Critically Ill Patients with Pre-existing Type 2 Diabetes) [15] to justify such a liberal glycemic control with patients with DM as is now underway.

It should be also noted that HbA1c level at ICU admission is significantly associated with worsened outcomes [16, 17]. According to these facts and our findings, to measure the HbA1c-ope might be relevant as a surrogate marker of premorbid glucose control and predictive value for outcomes.

It should be noted that HbA1c level is influenced by a number of factors. Our results and prior study [11] suggest that HbA1c levels may be influenced by intensive glycemic control and transfusion of red blood cells in acute ill setting. In patients after red cell transfusion, if stored serum samples collected before transfusion are available, it might be better to use those samples to estimate chronic glycemic control. In patients after intensive insulin control, the record of actual subsequent blood glucose level, not the HbA1c level, should be used as a surrogate of preoperative glycemic control.

#### Limitations

This study has some limitations. First, this was an observational study in nature, and thus our findings may have a potential bias. Second, this was a small single-center study with weak generalizability. Thus, our findings should be validated outside our study site. Finally, this study was conducted for 7 years due to the small number of patients with measurements of two HbA1c levels that were required for our study. Although the same technique was used to measure HbA1c levels during the study period, such a long period might skew the results.

We should note that our study may be relevant as first preliminary study to generate the hypothesis for the estimation of premorbid chronic glycemic control using the HbA1c level on the day of the operation in patients requiring emergency surgery. However, considering the limitations of our study, a multicenter prospective study should be necessary to confirm or refute our findings.

## Conclusions

In current study, there was a significant correlation between HbA1c levels on the day of an emergency operation and those measured within 30 days prior to the operation. The results also showed that a bias between the two HbA1c levels may be caused by short-term intensive insulin therapy or blood transfusion prior to emergency operation. Our findings suggest that HbA1c measured on the day of an emergency operation can be used to estimate previous glycemic control with an acceptable degree of accuracy, enabling personalized glycemic control in the perioperative period.

## Additional file

Additional file 1: The dataset used in current study. The date of operation and sex have been removed, and include age in ranges in order to help protect participant anonymity. (XLSX 12 kb)

## Abbreviations

ASA: American Society of Anesthesiology
CI: Confidential interval
DM: Diabetes mellitus
HbA1c-ope: HbA1c level measured on the day of the emergency operation
HbA1c-pre: HbA1c level measured within 30 days before the operation
ICU: Intensive care unit
